# *In Silico* Prediction and Bioactivity Evaluation of Chemical Ingredients Against Influenza A Virus From *Isatis tinctoria* L

**DOI:** 10.3389/fphar.2021.755396

**Published:** 2021-12-07

**Authors:** Chuipu Cai, Lvjie Xu, Junfeng Fang, Zhao Dai, Qihui Wu, Xiaoyi Liu, Qi Wang, Jiansong Fang, Ai-Lin Liu, Guan-Hua Du

**Affiliations:** ^1^ Division of Data Intelligence, Department of Computer Science, Key Laboratory of Intelligent Manufacturing Technology of Ministry of Education, College of Engineering, Shantou University, Shantou, China; ^2^ Science and Technology Innovation Center, Guangzhou University of Chinese Medicine, Guangzhou, China; ^3^ School of Basic Medical Sciences, Guangzhou University of Chinese Medicine, Guangzhou, China; ^4^ Institute of Materia Medica, Chinese Academy of Medical Sciences and Peking Union Medical College, Beijing, China; ^5^ The First Affiliated Hospital, Guangzhou University of Chinese Medicine, Guangzhou, China

**Keywords:** network-based identification, influenza A virus, virtual screening, *Isatis tinctoria* L., drug discovery

## Abstract

Influenza A virus (IAV) is one of the major causes of seasonal endemic diseases and unpredictable periodic pandemics. Due to the high mutation rate and drug resistance, it poses a persistent threat and challenge to public health. *Isatis tinctoria* L. (Banlangen, BLG), a traditional herbal medicine widely used in Asian countries, has been reported to possess strong efficacy on respiratory viruses, including IAV. However, its effective anti-IAV components and the mechanism of actions (MOAs) are not yet fully elucidated. In this study, we first summarized the chemical components and corresponding contents in BLG according to current available chemical analysis literature. We then presented a network-based *in silico* framework for identifying potential drug candidates against IAV from BLG. A total of 269 components in BLG were initially screened by drug-likeness and ADME (absorption, distribution, metabolism, and excretion) evaluation. Thereafter, network predictive models were built *via* the integration of compound–target networks and influenza virus–host proteins. We highlighted 23 compounds that possessed high potential as anti-influenza virus agents. Through experimental evaluation, six compounds, namely, eupatorin, dinatin, linarin, tryptanthrin, indirubin, and acacetin, exhibited good inhibitory activity against wild-type H1N1 and H3N2. Particularly, they also exerted significant effects on drug-resistant strains. Finally, we explored the anti-IAV MOAs of BLG and showcased the potential biological pathways by systems pharmacology analysis. In conclusion, this work provides important information on BLG regarding its use in the development of anti-IAV drugs, and the network-based prediction framework proposed here also offers a powerfulful strategy for the *in silico* identification of novel drug candidates from complex components of herbal medicine.

## Introduction

Influenza (flu) is an acute respiratory viral infection that leads to the continual emergence of seasonal epidemics and occasional global pandemics in humans, causing significant morbidity and mortality worldwide (Krammer et al., 2018). Influenza A virus (IAV), as one of the most contagious viruses among the influenza types A, B, and C, has always been a huge threat to public health that causes about 200,000 hospitalizations and 30,000 deaths per year (Xu et al., 2020). More importantly, a recent study has demonstrated that IAV possesses clear auxo-action on severe acute respiratory syndrome coronavirus 2 (SARS-CoV-2) infection, which could boost viral entry into cells and increase the viral load, causing worse lung damage in virus-infected mice. These results emphasize the great importance of influenza prevention, particularly in the context of the ongoing coronavirus disease-2019 (COVID-19) pandemic. One important reason for the epidemiological “success” of IAV is the highly variability owing to the constant production of new viral strains (Xu et al., 2020). The rapid evolution of influenza viruses reduces the effectiveness of current antiviral agents, which is thought to be the main bottleneck in antiviral treatment. Owing to the emergence of drug resistance of conventional anti-influenza drugs, such as inhibitors of neuraminidase (NA), the M2 ion channel, and RNA-dependent RNA polymerase (RdRp), the need for the development of anti-IAV drugs with novel modes of action is highly urgent (Zu et al., 2015; Xu et al., 2020).

Natural products isolated from herbal medicine possess chemical diversity and promiscuous target profiles, which are emerging as invaluable chemical resources for drug discovery (Shen, 2015; Cai et al., 2021). *Isatis tinctoria* L. (Banlangen, BLG) is a herbal medicine widely applied as regular seasonal influenza treatment in Asian countries (Wu et al., 2020). Previous experiments have showcased that the extracts of BLG possess broad-spectrum inhibitory activity against human influenza viruses (Yang et al., 2012; Li et al., 2017). BLG exerts immunoregulatory effects *in vitro* and *in vivo* and seems to combat viral infection by simultaneously targeting the host and the virus, which has obvious advantage over the chemical synthesis drugs on the market (Shin et al., 2010; ZiFeng et al., 2015). However, due to the complexity of the chemical components and the intricate target interactions, the active components and molecular mechanisms of BLG against influenza remain mostly unknown. Although previous studies integrating high-performance liquid chromatography (HPLC) analysis had preliminarily investigated the major chemical components in BLG, as summarized in Table 1, it is difficult to determine the exact ingredients that exert the anti-IAV effect. In addition, some trace ingredients may also have strong potency, although they may not be detected by HPLC. Hence, it is of great significance to excavate the potential medicinal ingredients from BLG with a novel efficient strategy.

**TABLE 1:** Information on the chemical analysis using the high-performance liquid chromatography (HPLC) method for the herb *Isatis tinctoria* L. (Banlangen, BLG).

Ref. (PMID)	Method	Sample Form	Component	Contents
28894621	RP-HPLC	Granules	R-goitrin	Mean = 0.162 (mg/g)
			S-goitrin	Mean = 0.127 (mg/g)
19160787	RP-HPLC	Crude drug	Epigoitrin	4.243 (mg/g)
		Granules		0.412 (mg/g)
29844266	HPLC-UV-CD	Crude drug	Progoitrin	Mean ± SD = 1.71 ± 1.99 (mg/g)
			Epiprogoitrin	Mean ± SD = 3.05 ± 3.16 (mg/g)
			R-goitrin	Mean ± SD = 0.18 ± 0.06 (mg/g)
			S-goitrin	Mean ± SD = 0.09 ± 0.03 (mg/g)
		Decoction pieces	Progoitrin	Mean ± SD = 0.81 ± 1.06 (mg/g)
			Epiprogoitrin	Mean ± SD = 1.57 ± 1.92 (mg/g)
			R-goitrin	Mean ± SD = 0.22 ± 0.14 (mg/g)
			S-goitrin	Mean ± SD = 0.12 ± 0.07 (mg/g)
		Granules	R-goitrin	Mean ± SD = 0.12 ± 0.15 (mg/g)
			S-goitrin	Mean ± SD = 0.06 ± 0.07 (mg/g)
32288995	HPLC–DAD–ESI/MS	Dry raw material	Cytidine	Mean ± SD = 0.24 ± 0.10 (mg/g)
			Uridine	Mean ± SD = 0.37 ± 0.13 (mg/g)
			Adenine	Mean ± SD = 0.07 ± 0.04 (mg/g)
			Guanosine	Mean ± SD = 0.34 ± 0.17 (mg/g)
			R,S-Goitrin	Mean ± SD = 1.65 ± 1.09 (mg/g)
			Adenosine	Mean ± SD = 0.31 ± 0.16 (mg/g)
22942750	UPLC-PDA	Prepared slices	Hypoxanthine	Mean ± SD = 0.08 ± 0.11 (mg/g)
			Uridine	Mean ± SD = 0.35 ± 0.07 (mg/g)
			Progoitrin	Mean ± SD = 2.52 ± 1.54 (mg/g)
			Epiprogoitrin	Mean ± SD = 2.68 ± 1.70 (mg/g)
			Adenosine	Mean ± SD = 0.36 ± 0.06 (mg/g)
			Guanosine	Mean ± SD = 0.4 ± 0.07 (mg/g)
			R,S-goitrin	Mean ± SD = 0.64 ± 0.19 (mg/g)
			Luconapin	Mean ± SD = 1.35 ± 1.21 (mg/g)
			Hypoxanthine	Mean ± SD = 8.38 ± 4.58 (mg/g)
		Crude herbs	Hypoxanthine	Mean ± SD = 0.04 ± 0.01 (mg/g)
			Uridine	Mean ± SD = 0.07 ± 0.01 (mg/g)
			Progoitrin	Mean ± SD = 5.86 ± 1.1 (mg/g)
			Epiprogoitrin	Mean ± SD = 6.25 ± 0.4 (mg/g)
			Adenosine	Mean ± SD = 0.12 ± 0.01 (mg/g)
			Guanosine	Mean ± SD = 0.16 ± 0.08 (mg/g)
			R,S-goitrin	Mean ± SD = 0.08 ± 0.02 (mg/g)
			Luconapin	Mean ± SD = 8.39 ± 1.86 (mg/g)
			Hypoxanthine	Mean ± SD = 20.96 ± 2.44 (mg/g)
16884885	LC-APCI-MS	Granules (root)	Tryptanthrin	Mean ± SD = 0.33 ± 0.20 (μg/g)
			Indigo	Mean ± SD = 1.01 ± 0.79 (μg/g)
			Indirubin	Mean ± SD = 0.95 ± 0.85 (μg/g)

The contents of the compounds were obtained from previously published chemical analysis literature of *Isatis tinctoria* L. and unified as average values*.* Detailed information on the HPLC methods and the detection results can be found in the original papers.

Nowadays, *in silico* approaches have been successfully applied in drug discovery and have dramatically facilitated efficiency and reduced costs (Cai et al., 2017). Structure-based and ligand-based approaches, such as the quantitative structure–activity relationship (QSAR) model, pharmacophore model, molecular docking, and similarity searches, are extensively used. However, ligand-based methods are always encumbered by the diversity and quality of the sample, while structure-based methods have the drawbacks of a slow computing speed and lack of crystal structures. Besides, these approaches ignore the inherent synergistic interactions between drugs and multiple therapeutic targets. Recently, network-based methods inspired by systems pharmacology, which comprehensively consider the information of the drug–target network and disease-related genes, provide new insights into the identification of active ingredients from the complex system of herbal medicine (Fang et al., 2017a; Wu et al., 2020). For instance, compound–target networks of natural products integrated with wide-scale genomic profiles of triple-negative breast cancer (TNBC) revealed wogonoside as a potent angiogenesis inhibitor for TNBC therapy (Huang et al., 2019). Researchers have built network-based models to infer the potential therapeutic relationships of natural products on coronary artery disease (Fang et al., 2019). Recently, we have identified 49 natural products from over 500 traditional Chinese herbs that have huge potential for cancer immunotherapy, and the success rate reached up to 65.31% through validation with published clinical and experimental evidence (Cai et al., 2021). Overall, network-based approaches of drug discovery offer effective strategies for discovering potential antiviral candidates against IAV.

**FIGURE 1:**
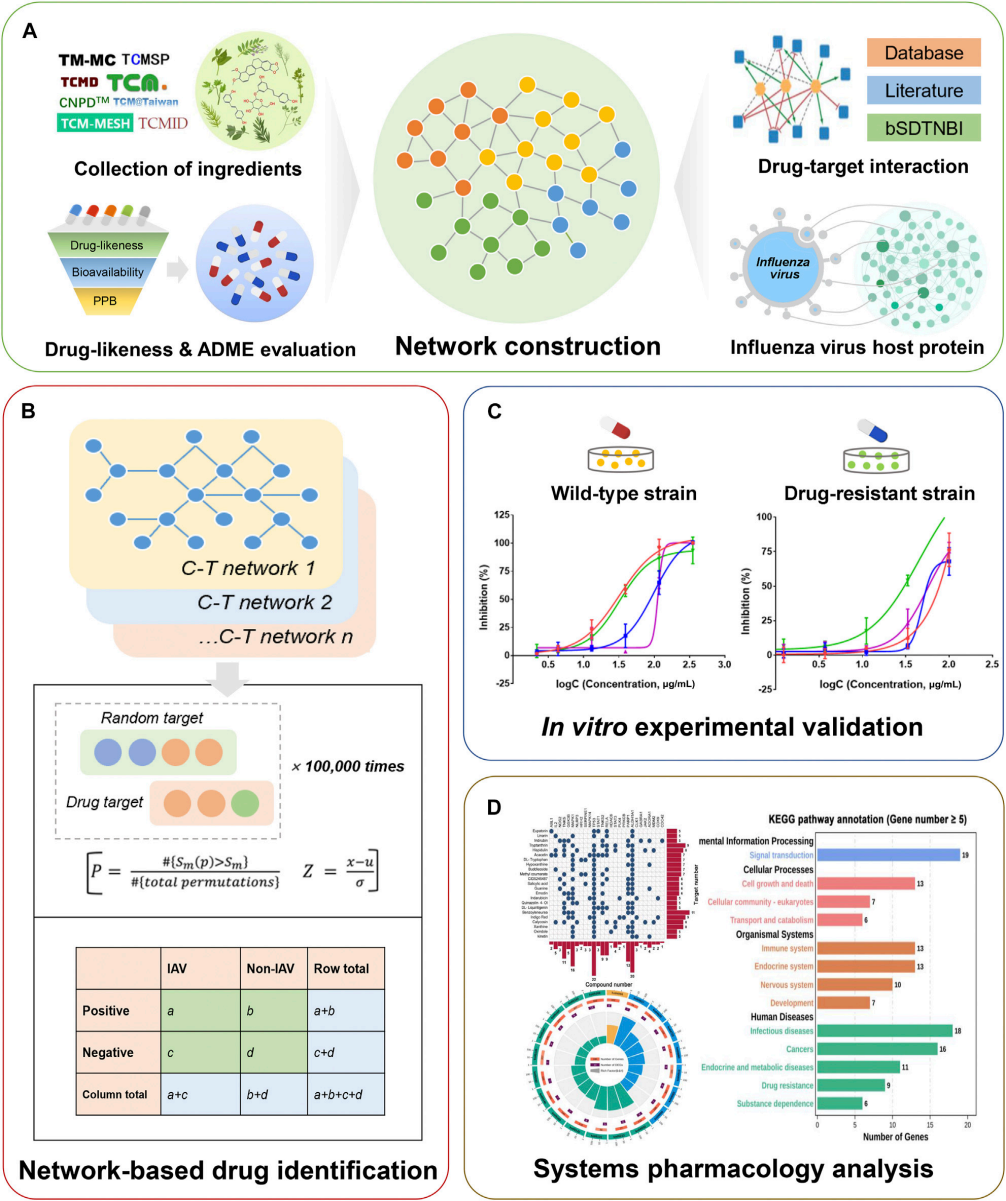
Schematic diagram illustrating the network methodology for the *in silico* identification of drug candidates against influenza A virus (IAV) from *Isatis tinctoria* L. (BLG). **(A)** Construction of the compound–target (C–T) networks of BLG. Ingredients screened by drug-likeness and ADME (absorption, distribution, metabolism, and excretion) evaluation, multi-source drug–target interactions, and influenza virus host proteins were integrated into the C–T network. **(B)** *In silico* identification of IAV drug candidates using network-based predictive models. Associations between the subnetwork of compounds and the influenza virus host protein set were calculated. **(C)** *In vitro* evaluation of potential anti-IAV candidates through cytopathic effect reduction assay on wild-type and drug-resistant virus strains. **(D)** Systems pharmacology-based exploration of the anti-IAV mechanism of actions (MOAs) of BLG.

In this work, we developed an *in silico* framework to identify potential drug candidates against IAV from BLG (Figure 1 and Supplementary Figure S1). We firstly collected comprehensive components of BLG from public databases and the chemical analysis literature. Subsequently, machine learning models were applied for the initial screening of the drug-likeness and ADME (absorption, distribution, metabolism, and excretion) properties of chemicals. Subsequently, compound–target (C–T) networks were constructed by consolidating computationally predicted and experimentally validated compound–target interactions (CTIs) and influenza virus host proteins. We further built network-based predictive models *via* the integration of the C–T network and influenza virus host protein set for identifying potential anti-IAV candidates. Additionally, we performed *in vitro* experimental assays to evaluate the inhibitory activity of the predicted positive compounds on wild-type and resistant strains of IAV (H1N1 and H3N2). Finally, the potential anti-IAV mechanism of actions (MOAs) of BLG, including key regulatory proteins, molecular functions, and biological pathways, were discussed *via* network analysis and gene enrichment.

## Materials and Methods

### *In Silico* Experiment

#### Collection and Structure Clustering of Ingredients in BLG

The constituent compounds of BLG were manually extracted from previously published chemical analysis literature (Table 1) and the following publicly available herbal medicine databases (accessed in February 2020, unless otherwise indicated): 1) TCM-MESH (Zhang et al., 2017); 2) Traditional Chinese Medicine database (TCMDb); 3) Traditional Chinese Medicine Integrated database (TCMID; accessed December 2018) (Huang et al., 2018); 4) Traditional Chinese Medicine database@Taiwan (TCM@Taiwan) (Chen, 2012); 5) TM-MC (Kim et al., 2015); 6) Traditional Chinese Medicine Systems Pharmacology (TCMSP) (Ru et al., 2014); and 7) Database of Traditional Chinese Medicine on Immuno-Oncology (TCMIO; accessed January 2021) (Liu et al., 2020). The names of compounds were standardized according to the PubChem database, while structures were converted to canonical SMILES and InChiKey formats using OpenBabelGUI (version 3.0.0) (O’boyle et al., 2011). After removing duplicate compounds with identical structures, a total of 269 compounds were retained for further study (Supplementary Table S1).

To investigate the chemical features of the ingredients in BLG, we performed clustering analysis for the 269 compounds. The chemical clustering analysis was conducted by calculating the root mean square value of the Tanimoto distance of pairwise compounds based on FCFP_6 fingerprint. Finally, the chemical scaffolds of the 269 ingredients were clustered into five chemical groups, and the structures of each cluster center were obtained.

#### Machine Learning Models for Drug-Likeness and ADME Screening

The machine learning models for drug-likeness and ADME screening were provided by the work of Jie et al. (2018). Specifically, the drug-likeness model was trained based on 6,731 drugs from the DrugBank database (Wishart et al., 2017) as positive samples and 6,769 molecules from the CHEMBL database (Anna et al., 2017) as negative ones. The model constructed by random forest (RF) with molecular descriptors from MACCS (Molecular Access System) achieved the best performance, with an accuracy of 0.801 on fivefold cross-validation. For the ADME models, the sample sources included previous literature and the DrugBank database. The models were built based on different machine learning algorithms, such as RF, support vector machine (SVM), recursive partitioning regression (RP), partial least square (PLS), naive Bayes (NB), and decision tree (DT), while seven types of descriptors (2D, Estate, MACCS, ECFP2, ECFP4, ECFP6, and FP2) were used to represent the molecular properties and structural information. Subsequently, the best model for each property was selected after comprehensive assessment. In this study, the models of human oral bioavailability and plasma protein binding (PPB) were applied to evaluate the chemical properties of absorption and distribution, respectively.

#### Construction of Compound–Target Networks for BLG

We constructed global C–T network and subnetworks for the ingredients in BLG by integrating both experimentally validated and computer-predicted CTIs. The experimentally validated targets contained both physical binding (direct) and functional (indirect) targets. Among them, the direct targets (*K*_*i*_/*K*_d_/IC_50_/EC_50_ < 10 μm) were extracted from BindingDB (Gilson et al., 2016) and ChEMBL (v21) (Anna et al., 2017), while the indirect targets were gathered from the Herbal Ingredients’ Targets (HIT) database (Hao et al., 2011), STITCH (Search Tool for Interactions of Chemicals) (Damian et al., 2016), and TCMID 2.0 (Huang et al., 2018). These databases were accessed in December 2018. We further supplemented the CTIs of BLG from our previously curated natural product–target interaction dataset, which covers over 2,000 natural product-related pharmacological academic papers (dating from January 2009 to December 2017). Only targets with standard UniProt accession number or belonging to *Homo sapiens* were retained.

In order to enrich the CTI network, computationally putative targets predicted by the balanced substructure–drug–target network-based inference (bSDTNBI) algorithm were imported. The method could prioritize potential targets for natural products using resource diffusion processes for the substructure–drug–target network (Fang et al., 2017b). In this study, the substructure items of each compound were calculated using the molecular fingerprint Klekota–Roth from PaDEL-Descriptor (version 2.18) (Yap, 2011). The parameters *α*, *β*, *γ*, and *κ* were set as 0.1, 0.1, −0.5, and 2, respectively. Parameter *α* was utilized to control the initial resource allocation of various node types, *β* was applied for the adjustment of the weighted values of different edge types, *γ* was imported to balance the effect of hub nodes in the process of resource diffusion, and *κ* represents the number of resource diffusion processes. The final generated predictive model showed satisfactory performance, with an area under the receiver operating characteristic curve (AUC) value of 0.958 ± 0.005 in 10-fold cross-validation (Fang et al., 2017b). Finally, the top 50 putative targets of each ingredient in BLG were obtained.

#### Manual Curation and Integration of Influenza Virus Host Protein Set

We comprehensively searched the published literature to obtain the influenza virus host proteins. The names of the collected proteins/genes were converted into unified gene symbol names and Entrez ID according to the NCBI Gene Database (https://www.ncbi.nlm.nih.gov/gene) and the Universal Protein Resource (https://www.uniprot.org/; Consortium, 2019). These proteins/genes were further consolidated into the influenza virus host protein set after removing duplicates. In total, the influenza virus host protein gene set consisted of 175 proteins (Supplementary Table S2).

#### Network-Based Statistical Models for the Identification of Anti-IAV Drugs

In this study, network-based models were constructed to measure the statistical correlation between each C–T network of ingredients in BLG and the influenza virus host protein gene set. We hypothesize that a compound has a higher possibility of being an anti-IAV drug if its regulatory target network is more likely to map onto the influenza virus host protein set. The null hypothesis asserts that the targets of a compound are randomly located at the gene profiles of the influenza virus host proteins across the human proteome. Here, we applied two types of statistical methods to build the network predictive models. The first model (model A) was constructed based on permutation test, as given by Eq. 1.

$$P=\frac{\#\left\{sm\left(p\right)>sm\right\}}{\#\left\{\;totalpermutations\right\}}$$ (1)

We randomly selected 175 genes, which is equal to the number of genes in the influenza virus host protein gene set from the protein profiles at the human genome-wide scale (covering 20,462 human protein-coding genes) from the NCBI database (Sayers et al., 2018). For each compound of BLG (drug candidate), a nominal *P* was computed by counting the number of permutations (Sm(*p*)) larger than the observed influenza virus host protein genes (Sm). The permutations were repeated 100,000 times and the resulting *p*-values obtained from the permutation tests. A *Z*-score was computed for each compound to be prioritized as potential drug candidates for influenza virus during the permutation test (Eq. 2).

$$Z=\frac{x-\mu }{\sigma }$$ (2)

where x is the actual number of influenza virus host proteins targeted by a given drug candidate, μ is the average number of influenza virus host proteins targeted by a given drug candidate during 100,000 permutations, and σ is the standard deviation.

We further utilized Fisher’s exact test to generate the other network predictive model (model B). Fisher’s exact test is a statistical significance test examining the significance of the association (contingency) between two classifications. In this model, the statistical significance of the enrichment of influenza virus host proteins in the target profiles of each drug candidate were obtained using Fisher’s exact test. The Benjamini–Hochberg method (Benjamini and Hochberg, 1995) was used to correct the resulting *p*-values of the models, and the negative logarithm values of *q*(−Log10(*q*)) were obtained. We determined the ingredients with significant correlations with the influenza host proteins based on the compound–target network. A cutoff [adjusted *p*-value (*q*) threshold] of 0.05 was set to differentiate the predicted positive and negative anti-IAV drug candidates. For those compounds with *q* values lower than 0.05, the larger the value of the *Z*-score (model A) or −Log10(*q*) (model B), the higher the possibility of being anti-IAV drug candidates.

#### Gene Enrichment Analysis

Gene Ontology (GO) term enrichment analysis and Kyoto Encyclopedia of Genes and Genomes (KEGG) pathway annotation were performed to explore the mechanisms (Kanehisa and Goto, 2000). Firstly, the gene sets of interest were mapped to the terms in the GO or KEGG database and the gene numbers were computed for each term. A hypergeometric test was used to define the significantly enriched GO or KEGG terms for the given gene sets compared to the genome background (*H. sapiens*). False discovery rate (FDR) was utilized to correct the calculated *p*-values, while terms with FDR ≤ 0.05 were regarded as significant.

#### Network Visualization and Statistical Analysis

The network was visualized and analyzed with Gephi (v0.9.2; https://gephi.org/) (Bastian et al., 2009). The statistical analysis, which included permutation test, Fisher’s exact test, and hypergeometric test, was performed with R (v3.01; http://www.r-project.org/) and the Python platform (v3.2; http://www.python.org/), while the figure plots were mainly drawn using Perl, R, and Microsoft Office 2019.

### *In Vitro* Experiment

#### Cell Culture, Virus, and Compounds

Madin–Darby canine kidney (MDCK) cells were cultured in Dulbecco’s modified Eagle’s medium (DMEM) supplemented with 10% fetal bovine serum (FBS) in a humidified incubator containing 5% CO_2_ atmosphere at 37°C. Influenza virus A/PR/8/34 (H1N1), A/Minfang/151/2000 (H3N2), and A/HebeiXinhua/SWL1106/2017 (oseltamivir- and amantadine-resistant H1N1) were kindly donated by the National Institute for Viral Disease Control and Prevention, Chinese Center for Disease Control and Prevention. The viruses were propagated in 7-day-old embryonated chicken eggs and preserved at−80°C. The test compounds and the positive control drug zanamivir were purchased from TopScience Co. Ltd. (Rizhao, China) and Sigma-Aldrich (St. Louis, MO, United States).

#### Cytotoxicity Test

MDCK cells grown into a monolayer in a 96-well plate were washed once with serum-free medium. Subsequently, the cells were treated with the test samples at different concentrations ranging from 1 to 100 μg/ml for 48 h at 34°C in 5% CO_2_. A blank medium served as the control. The crystal violet staining method was applied for cell viability determination (Xiao et al., 2021). The maximal non-toxic concentration (TC_0_) and median toxic concentration (TC_50_) values were computed through plotting the calculated percent cell viability as a function of the compound concentration (Zu et al., 2012).

#### Cytopathic Effect Reduction Assay

Four different time points for drug administration were adopted in the experiments: i) pretreatment: influenza virus at TCID_50_ = 100 were administered to the cells 1 h after the pretreatment of six serial dilutions of the test samples; ii) simultaneous treatment: the test samples were administered to the cells along with the influenza virus strain; iii) posttreatment: the test samples were administered to the cells 1 h after the adsorption of the influenza virus; and iv) pre-incubation treatment: the influenza virus was pre-incubated with the test samples for 1 h before being administered to the cells.

The total volume of each well was 100 μl, and TC_0_ was utilized as the maximum concentration and diluted to six triple gradient concentrations. Zanamivir served as the positive control drug, while infection control without drugs was also considered. The plates were incubated for 48 h at 34°C in humidified 5% CO_2_. The cytopathic effect (CPE) was determined with the crystal violet staining method, and half maximal inhibitory concentrations (IC_50_) were calculated using the resulting spectrophotometric data. The experiment was repeated at least three times.

CPE inhibition% = (OD_sample_ − OD_model_)/(OD_normal_ − OD_model_) × 100%

## Results

### Drug-Likeness Screening and ADME Evaluation

**FIGURE 2:**
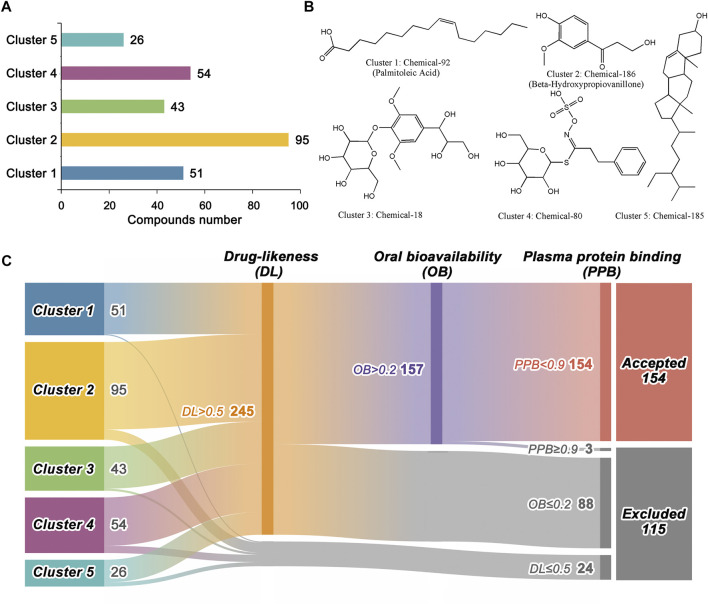
Chemical scaffold clustering analysis and preliminary screening of the 269 ingredients in *Isatis tinctoria* L. (BLG). **(A)** Statistics of the structures in the five cluster groups. Compounds with similar Tanimoto distance were clustered together. **(B)** Representative structures of the five cluster centers. **(C)** Drug-likeness and ADME (absorption, distribution, metabolism, and excretion) screening based on machine learning models. Detailed information is provided in Supplementary Table S1.

In total, 269 compounds from BLG were collected, which can be clustered into five groups according to their Tanimoto distance (Figure 2A). Cluster 2 contained the most compounds (*n* = 95), followed by cluster 4 (*n* = 54) and cluster 1 (*n* = 51). The structures of each cluster center are displayed in Figure 2B, which referred to the representative chemical scaffolds of the ingredients in BLG. Here, we evaluated the drug-likeness and ADME properties (PPB and human oral bioavailability) for initially excluding unsuitable candidates. Drug-likeness is utilized in drug design to describe how “druglike” a particular molecule is with respect to various molecular properties and structural features. As shown in Figure 2C, the classification model of drug-likeness eliminated 24 compounds with predicted positive probability lower than 50%, which left 245 compounds. Thereafter, oral bioavailability, one of the key pharmacokinetic indexes reflecting the efficiency of absorption, was further assessed. In this step, 88 compounds were predicted to have a bioavailability lower than or equal to 20%. PPB ability has significant effects on the pharmacodynamic action of a drug since one of the main mechanisms for drug uptake and distribution is through PPB. A compound with PPB <90% is considered suitable, while drugs with a high protein binding rate may have a low therapeutic index (Jie et al., 2018). During this process, three compounds were removed for inappropriate PPB property. Finally, 115 out of the 269 compounds in BLG were excluded, with the remaining 154 utilized for further study (Supplementary Table S1).

### Compound–Target Network Analysis of Ingredients in BLG

**FIGURE 3:**
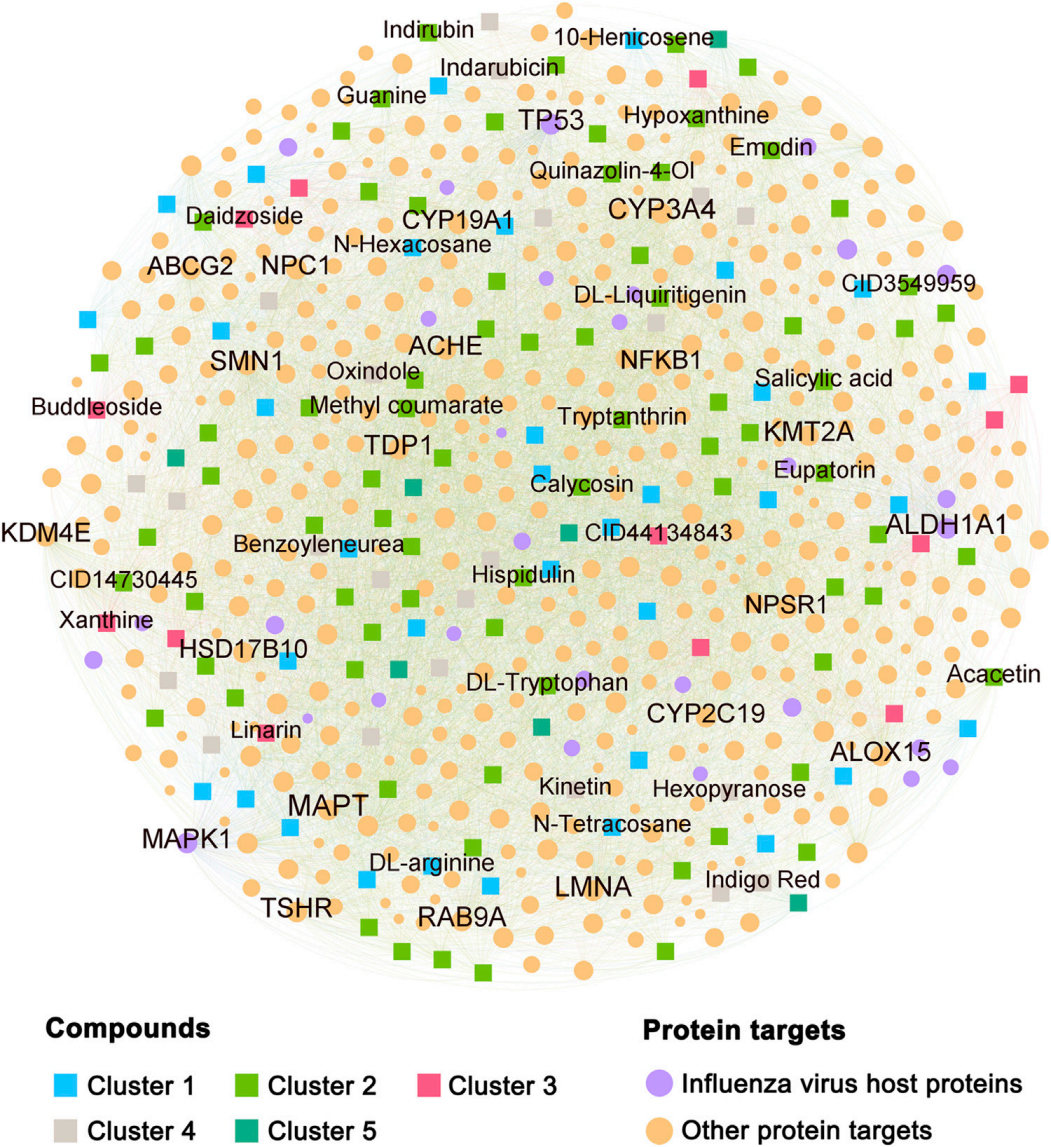
Compound–target (C–T) network for 154 candidates derived from *Isatis tinctoria* L. (BLG). The *font size of the label* and *node* is proportional to the degree (connectivity) of the item. *Squares* and *spots* in the network represent the compounds and protein targets, respectively. Compounds were classified according to chemical scaffold clustering analysis and displayed in *different colors*. Labels of the top 20 protein targets with the highest degree and compounds possessing at least five influenza virus host protein targets are displayed.

The C–T network was composed of interactions linking the screened compounds of BLG and their corresponding targets, which contained 7,845 CTIs connecting 154 compounds with 472 protein targets (Figure 3 and Supplementary Table S3). We further mapped the profiles of the influenza virus host proteins onto the C–T network and calculated the degree of each node. In total, there were 607 pairs of CTIs linked to 30 influenza-related targets. The average compound degree (*D*) of an influenza-related protein was 20.2, while the average influenza-related target degree (*K*) of a compound was 3.9. Among the top 20 targets with highest degree, ALDH1A1 (*D* = 150), MAPK1 (*D* = 149), and TP53 (*D* = 146) were influenza-related targets, indicating their important role in the anti-influenza effect of BLG. Interestingly, we found that each of the 154 compounds possessed at least one influenza virus host protein target. The top 5 compounds with the most influenza-related targets were acacetin (*K* = 11), hispidulin (*K* = 9), indigo red (*K* = 8), tryptanthrin (*K* = 8), and calycosin (*K* = 8). These compounds have been reported to exert potential as antiviral drug candidates against multiple viral infectious diseases, such as respiratory syncytial virus, severe acute respiratory syndrome (SARS), Middle East respiratory syndrome (MERS), COVID-19, human immunodeficiency virus-1 (HIV-1), coxsackie virus B3 (CVB3), and influenza–parainfluenza (Adhikari et al., 2021; Omrani et al., 2021; Mok et al., 2014; Chen et al., 2011; 2020; Mani et al., 2020). Overall, the C–T network revealed that the constituent components of BLG could exert synergistic anti-flu effects through targeting multiple influenza virus host proteins.

### Network-Based Identification of Potential Anti-IAV Ingredients From BLG

**FIGURE 4:**
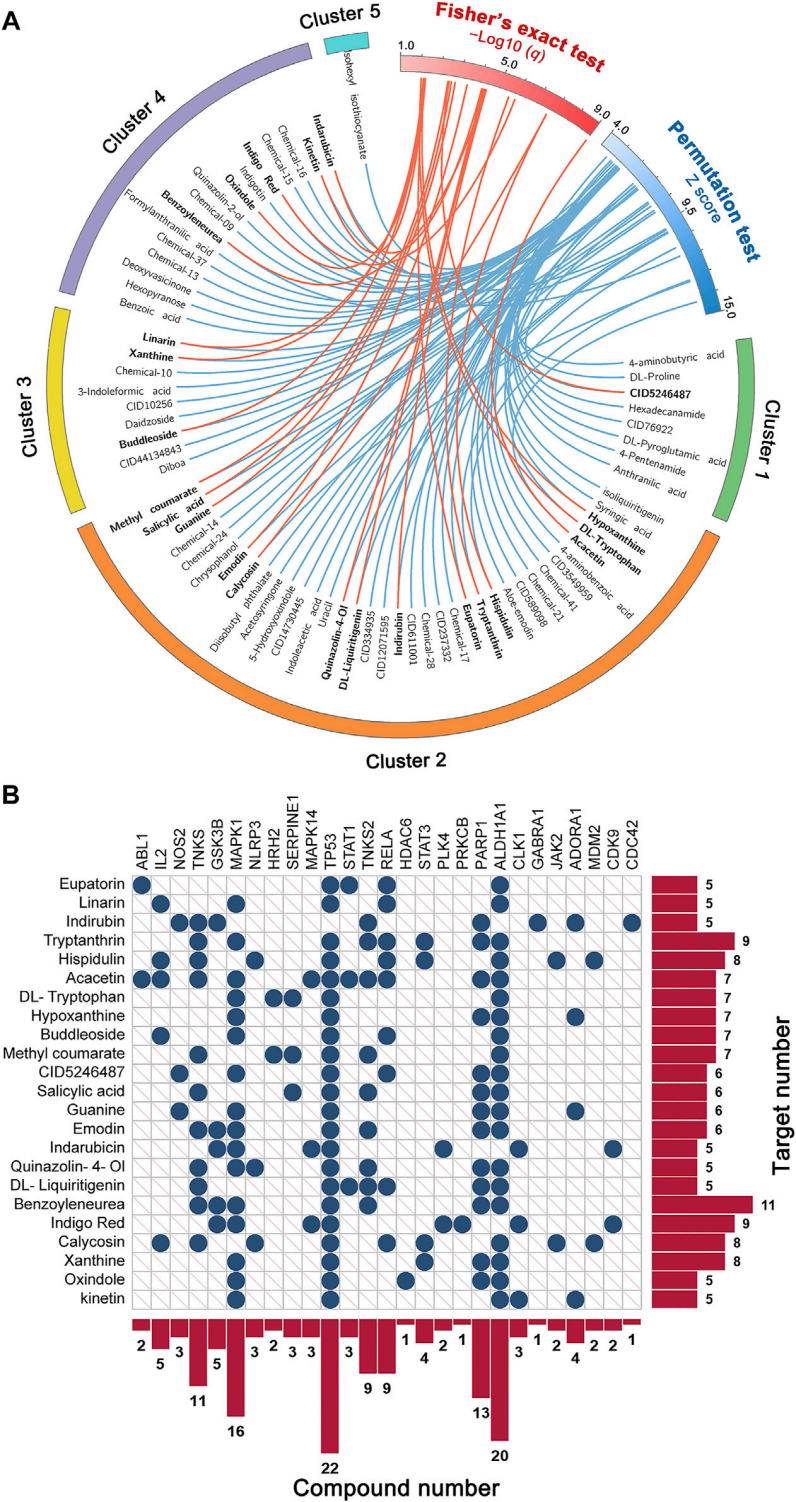
Identification of the potential anti-influenza A virus (IAV) candidates in *Isatis tinctoria* L. (BLG) using statistical network models. **(A)** Circos plot exhibiting the 71 predicted positive compounds (*q* < 0.05) by models based on permutation test (model A) and Fisher’s exact test (model B). Compounds simultaneously predicted as positive by the two models are highlighted in *bold font*. **(B)** Target distribution of the 23 compounds that were simultaneously identified as potential anti-IAV candidates by the two models.

Based on the C–T network of the 154 compounds and the influenza virus host protein set, two network predictive models were built to identify novel anti-IAV agents from BLG. By setting the threshold of *q* < 0.05, models A and B identified 71 and 23 compounds as potent active compounds, respectively (Figure 4A and Supplementary Table S4). To improve the prediction accuracy, we merged the prediction results of the two models and obtained an intersection of the two sets of predicted positive compounds. The 23 compounds that had been predicted as potential anti-IAV agents simultaneously could serve as more promising candidates (Figure 4B). Indeed, some of them exhibited broad-spectrum antiviral effects in previously published experiments. For instance, indirubin was found to possess concentration-dependent inhibitory activity on Japanese encephalitis virus (JEV) replicated *in vitro* and exhibited good protective effects in a mouse model with lethal JEV challenge (Chang et al., 2012). It has also been reported to decrease the H1N1 susceptibility and alleviate lung damage in a restraint-stressed mouse model by regulating the mitochondrial antiviral signaling pathway (Chong et al., 2017). In addition, tryptanthrin was confirmed to effectively inhibit the CPE and virus yield (IC_50_ = 1.52 μM) in HCoV-NL63-infected cells (Tsai et al., 2020). It also showed promise as a potential candidate against SARS-CoV-2 and other viruses (Mani et al., 2020; Xu et al., 2020). To sum up, evidence from literature preliminarily confirmed the reliability of the predicted results of network-based models, suggesting that the antiviral ability of these compounds against IAV deserves to be further experimentally validated, especially on resistant strains.

### Evaluation of the *In Vitro* Efficacy of Drug Candidates on IAVs

**FIGURE 5:**
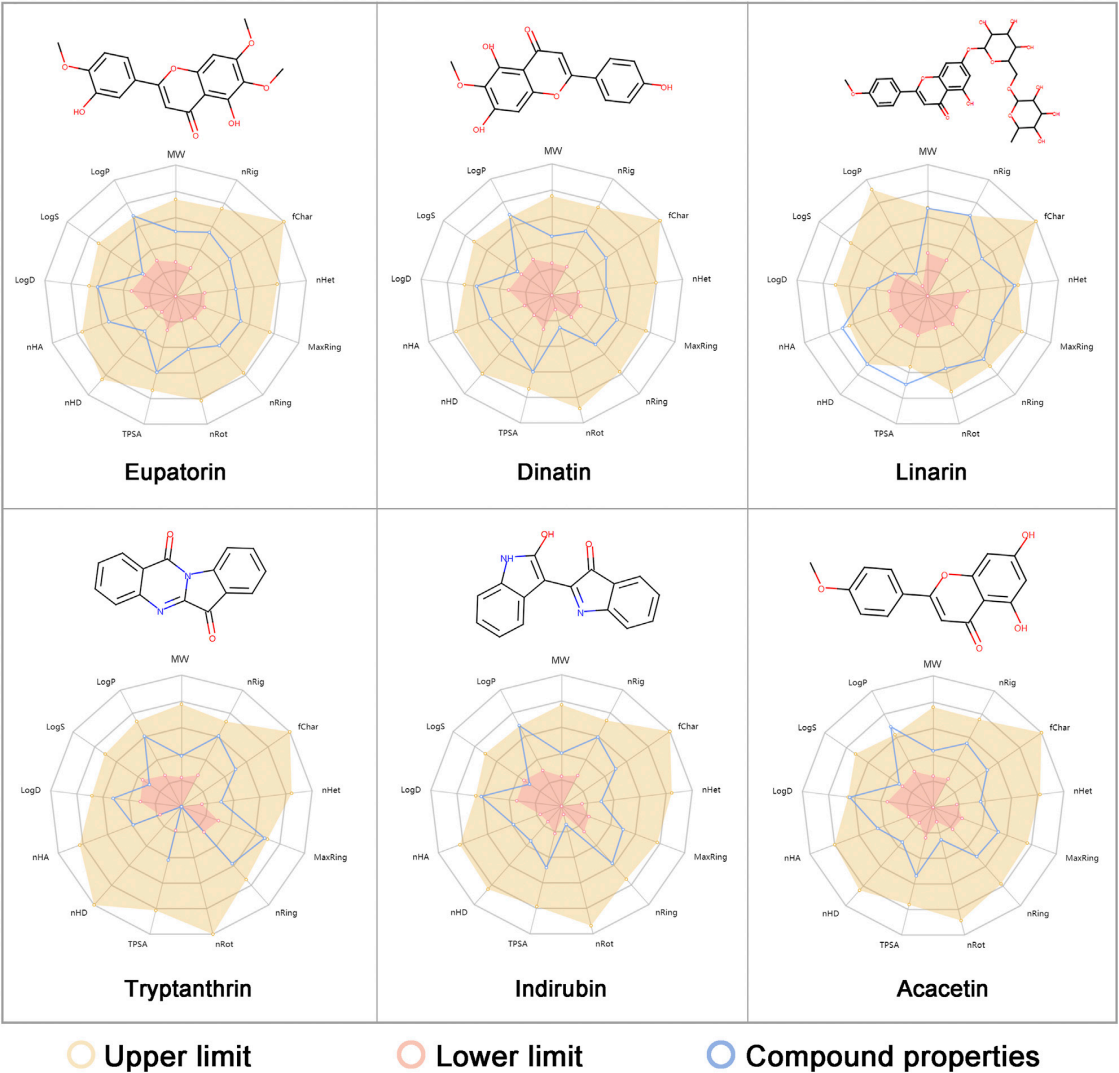
Compound structures and physicochemical properties of the six predicted anti-influenza A virus (IAV) ingredients (eupatorine, dinatin, linarin, tryptanthrin, indirubin, and acacetin) selected for *in vitro* evaluation. The radar plot exhibits the physicochemical properties of each compound (in *blue*) and the reference optimal scope (in *yellow* and *red*). The optimal range of the chemical and physicochemical properties is provided by ADMETlab 2.0 (Xiong et al., 2021). Detailed explanations of the endpoints can be found in Supplementary Table S5.

*In vitro* experimental evaluation was performed to further verify the *in silico* prediction of the potential anti-IAV candidates. By comprehensive considerion of the network-based predicted ranking, in terms of chemical structure, physicochemical property, availability, and accessibility, we finally selected the six most promising candidates from the 23 predicted compounds for evaluation of their anti-IAV efficacy: eupatorin, dinatin, linarin, tryptanthrin, indirubin, and acacetin (Figure 5). A cytotoxicity test was performed to determine whether the concentrations of the test samples used for the experiments would affect cell viability. The results showed that the TC_0_ and TC_50_ values of each compound were greater than 100 μg/ml.

CPE reduction assay was performed to evaluate the inhibition activity of the six compounds with two wild-type and one drug-resistant IAV strains: A/PR/8/34 (H1N1), A/Minfang/151/2000 (H3N2), and A/HebeiXinhua/SWL1106/2017 (oseltamivir- and amantadine-resistant H1N1). Our experiments included four different time points for drug administration: pretreatment, simultaneous treatment, posttreatment, and pre-incubation treatment assay. As listed in Table 2, all of the six compounds showed efficacy (selection index, SI > 1) against the wild-type strain A/PR/8/34 (H1N1). When the H1N1 virus was treated simultaneously or pre-incubated with the test compounds, these candidates tended to have stronger antiviral activity compared with other action modes, suggesting that they may directly suppress the H1N1 activity or impair viral adsorption. For the wild-type strain A/Minfang/151/2000 (H3N2), all the drug candidates except for dinatin also showed antiviral effects in the CPE reduction assay (Table 3). Among them, acacetin showed the best inhibitory activity with an IC_50_ of 7.62 ± 1.45 µg/ml when it was added to the cells before virus infection, which indicates its potential anti-flu prophylactic effect.

**TABLE 2:** Cytopathic effect (CPE) reduction assay results of the six predicted compounds toward the wild-type strain A/PR/8/34 (H1N1)

Compound	Pretreatment		Simultaneous treatment		Posttreatment		Pre-incubation treatment	
	IC_50_	SI	IC_50_	SI	IC_50_	SI	IC_50_	SI
Acacetin	7.62 ± 1.45	>13.12	9.22 ± 1.23	>10.85	11.34 ± 2.93	>8.82	20.41 ± 1.24	>4.9
Eupatorin	59.46 ± 3.58	>1.68	63.99 ± 3.38	>1.56	55.77 ± 3.86	>1.79	88.92 ± 7.34	>1.12
Dinatin	ND	ND	37.64 ± 2.29	>2.66	ND	ND	ND	ND
Linarin	63.22 ± 3.32	>1.58	54.27 ± 3.01	>1.84	72.21 ± 2.42	>1.38	56.26 ± 0.78	>1.78
Tryptanthrin	25.55 ± 2.19	>3.91	35.04 ± 2.98	>2.85	64.7 ± 3.57	>1.55	42.79 ± 0.59	>2.34
Indirubin	58.45 ± 10.23	>1.71	63.45 ± 5.27	>1.58	86.3 ± 5.68	>1.16	69.49 ± 1.36	>1.44
Zanamivir^a^	0.93 ± 0.16	>107.53	0.12 ± 0.03	>833.33	0.35 ± 0.09	>285.71	0.16 ± 0.03	>625

The experiments were conducted with four different time points for drug administration (pretreatment, simultaneous treatment, posttreatment, and pre-incubation treatment) and repeated three times. Detailed description of the drug administration mode can be found in *Materials and Methods*. Data are expressed as the mean ± SD (*n* = 3).

*IC*_*50*_, 50% effective concentration (in micrograms per milliliter); *ND*, not detected (IC_50_ > 100 μg/ml); *SI*, selection index (TC_50_/IC_50_)

^a^Positive control drug

**TABLE 3:** Cytopathic effect (CPE) reduction assay results of the six predicted compounds toward the wild-type strain A/Minfang/151/2000 (H3N2)

Compound	Pretreatment		Simultaneous treatment		Posttreatment		Pre-incubation treatment	
	IC_50_	SI	IC_50_	SI	IC_50_	SI	IC_50_	SI
Acacetin	7.7 ± 1.6	>12.99	13.62 ± 2.72	>7.34	9.21 ± 0.87	>10.86	9.41 ± 0.54	>10.63
Eupatorin	69.31 ± 1.97	>1.44	40.75 ± 1.15	>2.45	73.85 ± 1.34	>1.35	92.58 ± 0.33	>1.08
Dinatin	ND	ND	ND	ND	ND	ND	ND	ND
Linarin	81.85 ± 2.76	>1.22	41.96 ± 1.29	>2.38	86.35 ± 9.88	>1.16	77.71 ± 3.08	>1.29
Tryptanthrin	46.35 ± 2.05	>2.16	40.32 ± 1.81	>2.48	91.84 ± 13.1	>1.09	71.72 ± 0.44	>1.39
Indirubin	77.23 ± 6	>1.29	ND	ND	84.93 ± 5.09	>1.18	55.45 ± 2.03	>1.8
Zanamivir^a^	0.78 ± 0.29	>128.21	0.38 ± 0.05	>263.16	0.98 ± 0.14	>102.04	1.02 ± 0.28	>98.04

The experiments were conducted with four different time points for drug administration (pretreatment, simultaneous treatment, posttreatment, and pre-incubation treatment) and repeated three times. Detailed description of the drug administration mode can be found in *Materials and Methods*. Data are expressed as the mean ± SD (*n* = 3).

*IC*_*50*_, 50% effective concentration (in micrograms per milliliter); *ND*, not detected (IC_50_ > 100 µg/ml); *SI*, selection index (TC_50_/IC_50_)

^a^Positive control drug

**FIGURE 6:**
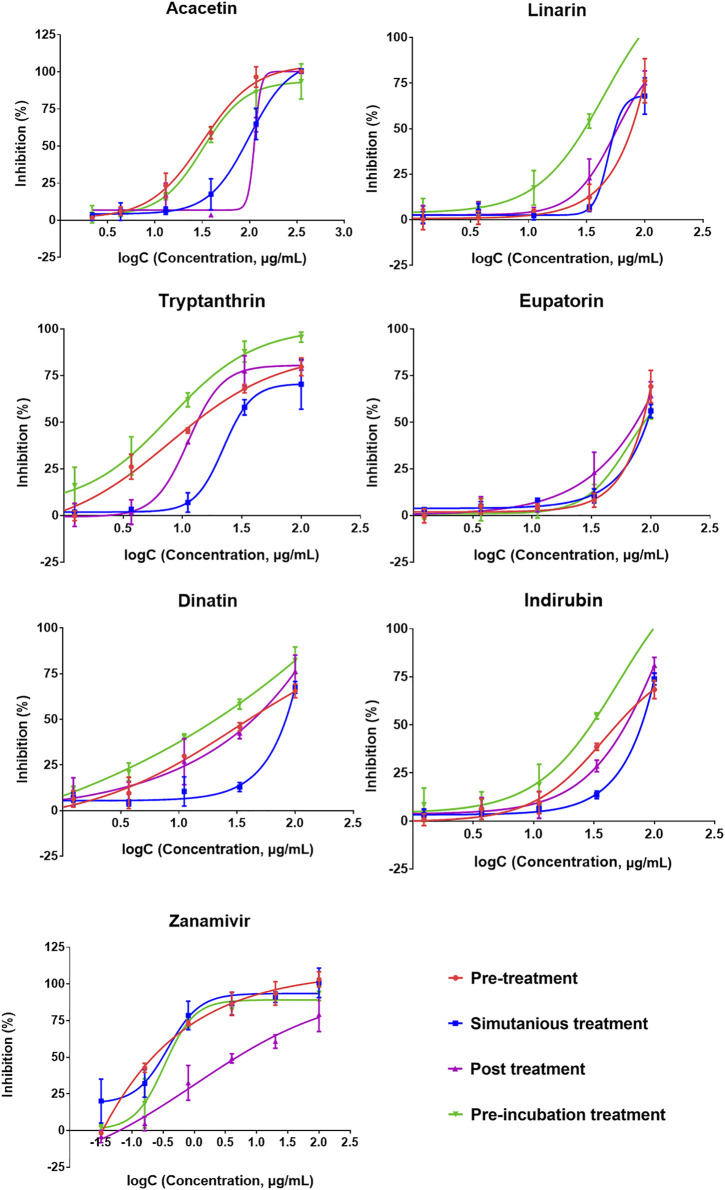
Cytopathic effect (CPE) reduction assay results of the six predicted compounds (eupatorin, dinatin, linarin, tryptanthrin, indirubin, and acacetin) and positive control drug (zanamivir) toward the oseltamivir- and amantadine-resistant H1N1. The 50% effective concentration (IC_50_) values are provided in Table 4.

We further evaluated whether the candidates are also effective against the drug-resistant strain. The results of the CPE reduction assay of the six predicted compounds toward A/HebeiXinhua/SWL1106/2017 (oseltamivir- and amantadine-resistant H1N1) are presented in Figure 6 and Table 4. As listed in Table 4, oseltamivir and ribavirin showed inefficacy in the four modes of drug administration tested for the resistant H1N1 strain, while all other tested candidates demonstrated inhibitory efficacy to some degree. Among them, acacetin and tryptanthrin exhibited stronger antiviral activity (Figure 6). Previous studies have shown their promising inhibitory effect against multiple viruses, such as coronavirus, respiratory syncytial virus, and influenza–parainfluenza viruses (Tsai et al., 2020; Adhikari et al., 2021). Additionally, according to the results of the drug administration modes (pretreatment, simultaneous treatment, and posttreatment), the earlier the samples were added to the cells, the better the protective effect they may possess on cells against the virus. Taking acacetin as an example, the average IC_50_ values for the drug-resistant IAV strain with pretreatment, simultaneous treatment, and posttreatment were 8.44 ± 0.39, 24.33 ± 2.44, and 25.81 ± 1.85 μg/ml, respectively. Correspondingly, its SI (TC_50_/IC_50_) decreased. Besides, acacetin (IC_50_ = 9.25 ± 0.87 μg/ml) and tryptanthrin (IC_50_ = 7.02 ± 0.65 μg/ml) also exhibited good activities with pre-incubation treatment, indicating that they may have a direct inhibition function on the drug-resistant H1N1 strain. Overall, the six predicted anti-IAV candidates exhibited inhibitory efficacy in the CPE reduction assay both on wild-type and drug-resistant strains and tended to possess better activity with the pretreatment drug administration mode. Although the action intensities of the candidates were lower than that of the positive control drug zanamivir, the preliminary anti-IAV activities suggested their high potential as hit compounds that deserve to be further optimized.

**TABLE 4:** Cytopathic effect (CPE) reduction assay results of the six predicted compounds toward the oseltamivir- and amantadine-resistant H1N1.

Compound	Pretreatment		Simultaneous treatment		Posttreatment		Pre-incubation treatment	
	IC_50_	SI	IC_50_	SI	IC_50_	SI	IC_50_	SI
Acacetin	8.44 ± 0.39	>11.85	24.33 ± 2.44	>4.11	25.81 ± 1.85	>3.87	9.25 ± 0.87	>10.81
Eupatorin	71.82 ± 6.58	>1.39	87.17 ± 5.23	>1.15	69.16 ± 2.12	>1.45	89.29 ± 5.22	>1.12
Dinatin	42.19 ± 4.61	>2.37	70.49 ± 3.85	>1.42	42.45 ± 1.57	>2.36	20.16 ± 1.28	>4.96
Linarin	64.67 ± 5.58	>1.55	73.93 ± 8.16	>1.35	58.57 ± 3.55	>1.71	29.24 ± 4.04	>3.42
Tryptanthrin	13.8 ± 0.47	>7.25	28.25 ± 2.04	>3.54	15.18 ± 0.58	>6.59	7.02 ± 0.65	>14.25
Indirubin	51 ± 4.05	>1.96	64.76 ± 1.67	>1.54	52.18 ± 0.87	>1.92	28.85 ± 0.22	>3.47
Oseltamivir	ND	ND	ND	ND	ND	ND	ND	ND
Amantadine	ND	ND	ND	ND	ND	ND	ND	ND
Zanamivir^a^	0.2 ± 0.03	>500	0.27 ± 0.09	>370.37	4.43 ± 1.23	>22.57	0.35 ± 0.12	>285.71

The experiments were conducted with four different time points for drug administration (pretreatment, simultaneous treatment, posttreatment, and pre-incubation treatment) and repeated three times. Data are expressed as the mean ± SD (*n* = 3). Detailed description of the drug administration mode can be found in *Materials and Methods*.

*IC*_*50*_, 50% effective concentration (in micrograms per milliliter); *ND*, not detected (IC_50_ > 100 µg/ml); *SI*, selection index (TC_50_/IC_50_)

^a^Positive control drug

### Exploration of the Molecular Mechanisms of BLG Against IAV

As the CPE reduction experiments exhibited, all six tested compounds showed inhibitory activity against IAV. The high hit rates further proved the reliability of our prediction and demonstrated the high potential as anti-IAV agents of the remaining 17 compounds that have not been experimentally validated yet. Hence, it is plausible to hypothesize that these 23 compounds predicted by the network models are the main active anti-IAV constituents of BLG. We next explored the synergistic effects of these compounds to showcase the underlying MOAs of BLG against IAV. As shown in Figure 4B, the 23 compounds acted on 27 influenza virus host protein targets. These compounds shared a lot of common protein targets, while each target was also targeted by multiple compounds. Among the protein targets, TP53 interacted with most compounds (*n* = 22), followed by ALDH1A1 (*n* = 20) and MAPK1 (*n* = 16). TP53 was identified as the most extensive essential host factor-interacting virus-targeting protein (EHF-interacting VTP) for IAV, which holds great potential in the discovery of host-directed antiviral agents (Xie et al., 2015). In addition, it was reported that ALDH1A1 downregulates and translocates to the nucleus from the cytoplasm during H3N2 virus infection, which may be associated with lipid metabolism that plays multifaceted roles in the life cycle of influenza virus and in virus–host interactions of (Chan et al., 2010; Heaton and Randall, 2011; Wu et al., 2013). The host protein kinase MAPK1 was also validated as essential for the replication of influenza virus (Bakre et al., 2013). These findings suggest the key regulatory targets of BLG for exerting its anti-IAV effects.

**FIGURE 7:**
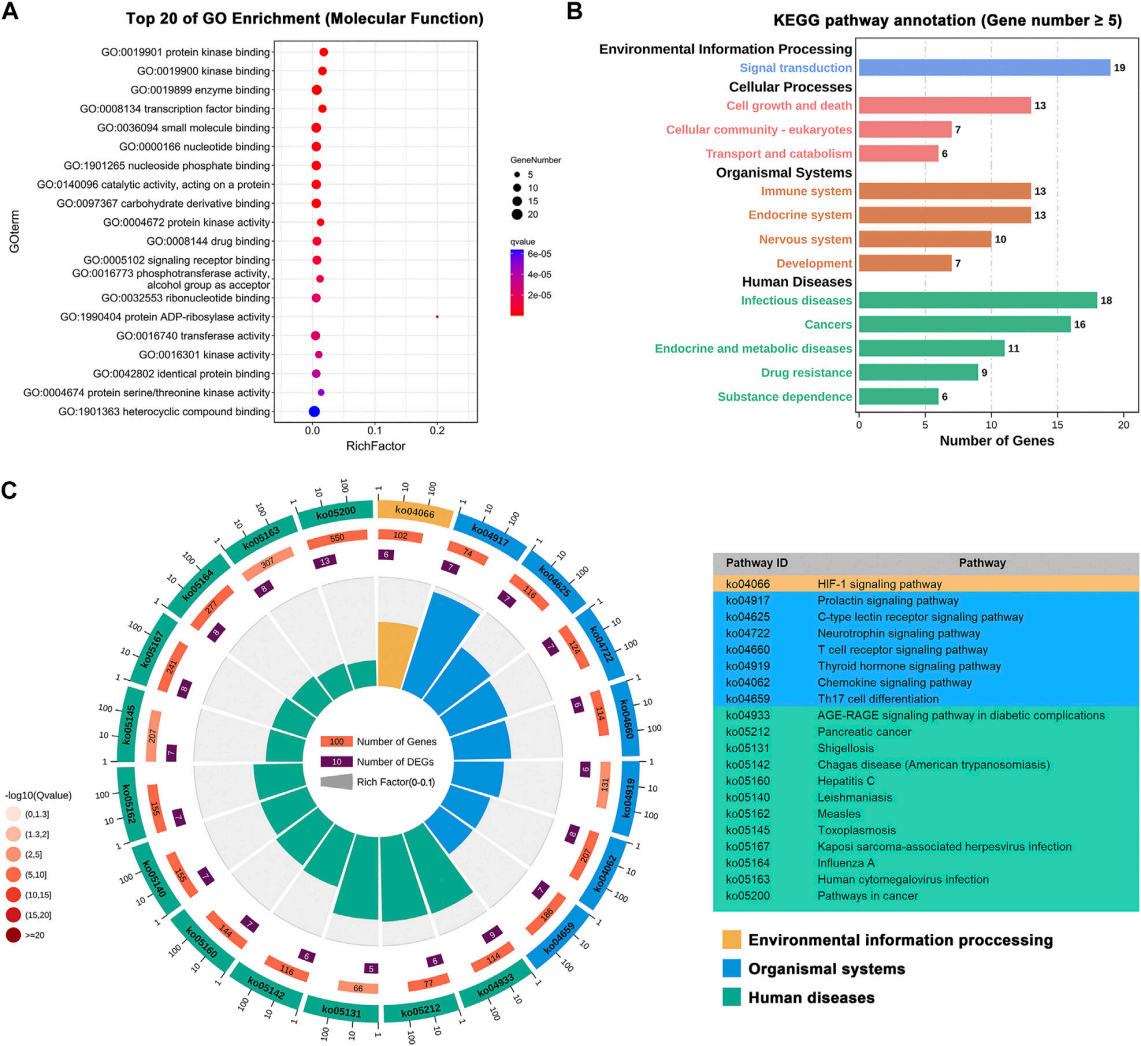
Exploration of the molecular mechanisms of *Isatis tinctoria* L. (BLG) against influenza A virus (IAV) through gene enrichment analysis. Gene Ontology (GO) and Kyoto Encyclopedia of Genes and Genomes (KEGG) annotations were performed on the 27 influenza virus host genes that may be regulated by the 23 predicted anti-IAV compounds in BLG identified by network analysis. **(A)** Top 20 molecular function terms in GO enrichment with the lowest *q* values. **(B)** KEGG pathway annotation. Different KEGG classes are displayed in *various colors*. **(C)** Top 20 terms in KEGG enrichment with the lowest *q* values.

Based on the 27 identified key targets, we further performed gene enrichment analysis, including GO term enrichment analysis and KEGG pathway annotation (Kanehisa and Goto, 2000), to explore the underlying molecular mechanisms of BLG against IAV. The top 20 molecular function terms in GO enrichment analysis are presented in Figure 7A. It is shown that these targets were involved in various basic molecular functions related to virus infection, such as the binding and activity of protein kinase, enzyme, and transcription factor. KEGG pathway annotation further elucidated the biological pathways that may be regulated by BLG (Supplementary Table S6). As shown in Figure 7B, most of these pathways were associated with virus infection. For instance, among the organismal system-related pathways, immune system is the largest group, with 13 pathways. It is universally acknowledged that respiratory virus infection usually induces effective immunity, including innate immune response and adaptive immune response, but overactive responses are correlated with pathophysiology (Stambas et al., 2020). In the category of human disease, 18 pathways are related to infectious diseases and 9 are correlated with drug resistance. Moreover, a lot of pathways are associated with signaling transduction, cell growth, and death. Figure 7C exhibits the top 20 enriched KEGG pathways with the highest *q* values. Accumulating pieces of evidence have demonstrated that these pathways are interconnected with the anti-influenza effect of drugs. For instance, the hypoxia inducible factor-1 (HIF-1) signaling pathway (ko04066) was one of the significantly enriched pathways with a *q* value of 6E−6. HIF-1α is an important factor in the development and repair of acute lung injury and could regulate glycolysis and AMPKα-ULK1-mediated autophagy, which ultimately affects IAV replication (Zhao et al., 2020). In addition, IAV infection is a complicated process, and its outcome is largely determined by the extensive release of chemokines and pro-inflammatory cytokines after its spread to the lung. Obviously, the chemokine signaling pathway (ko04062, *q* = 2E−6) plays an important role in this process (Zhang et al., 2020). A recent *in vitro* study has shown that the polysaccharides of BLG significantly decreased the expressions of chemokines stimulated by A/PR/8/34 (H1N1), including IP-10, MIG, and CCL-5 (Li et al., 2017). Taken together, the gene enrichment analysis highlighted the influenza-related molecular functions and biological pathways that merit attention, helping to understand the potential anti-IAV mechanisms of BLG.

## Discussion

Influenza is an acute respiratory infectious disease caused by influenza virus infection that brings serious threat and heavy burden to public health. As the most contagious influenza virus type, IAVs have always been the major cause of seasonal and pandemic influenza in humans. Owing to the extensive use of antiviral treatment, the problem of drug resistance has been increasing (Wu et al., 2020). For instance, in Europe, the number of seasonal H1N1 infections that are resistant to oseltamivir was up to 25% (Fiore et al., 2011). As an alternative treatment strategy, herbal medicine may significantly reduce the likelihood of emergence of viral resistance due to its heterogeneous ingredients and multiple target interactions (ZiFeng et al., 2015). *Isatis tinctoria* L. (BLG) is a popular anti-influenza virus herbal medicine widely used in China and other Asian countries. Previous *in vitro* and *in vivo* studies have proven that some of its components possess antiviral potential toward multiple influenza viruses (Yang et al., 2013; Su et al., 2016; Chen et al., 2021). However, to the best of our knowledge, there are only a few studies focusing on the comprehensive screening of promising compounds against IAV from BLG. In this work, we proposed a framework combining *in silico* prediction and *in vitro* evaluation to elucidate the effective substances and underlying molecular mechanisms of BLG against IAV (Figure 1 and Supplementary Figure S1). The framework consisted of the following aspects: 1) drug-likeness and ADME virtual screening based on robust machine learning models; 2) construction of a global C–T network of BLG *via* integration of literature-derived and computationally putative CTIs and high-quality influenza virus host protein gene set; 3) prioritization of the potential anti-IAV ingredients in BLG using network-based predictive models; 4) *in vitro* evaluation of the most promising predicted candidates for anti-IAV activity; and 5) exploration of the anti-IAV mechanism through systems pharmacology analysis including key target identification and gene enrichment.

Recently, several *in silico* studies have reported on the discovery of anti-influenza drugs. In these studies, the structure-based drug design strategy is the mainstream method. Molecular docking techniques were widely applied for the identification of small molecule inhibitors binding to influenza-related targets, such as polymerase acidic (PA) protein (Watanabe et al., 2017), hemagglutinin (Mathew et al., 2018), and nucleoprotein (Makau et al., 2017). However, these structure-based approaches have always only considered the information of a single drug target, while the complex relationship among drugs, multi-targets, and disease, which is precisely the pharmacological characteristic of herbal medicine, has been ignored. Compared to previous studies, the framework proposed in this study showcased several advantages. Firstly, the *in silico* approach applied here took into account both the chemical properties and the drug–target interactions. We applied statistical methods, including permutation test and Fisher’s exact test, to build predictive models based on the C–T network and influenza virus–host proteins, which significantly narrowed down the study scope of the ingredients in BLG and facilitated the identification of potential anti-IAV candidates. Secondly, our predictive models showed good applicability and accuracy. We identified 23 potential active ingredients that may exert major anti-IAV effects in BLG, of which six (eupatorin, dinatin, linarin, tryptanthrin, indirubin, and acacetin) were confirmed to have inhibitory activity both on wild-type and drug-resistant strains of IAV (H1N1 and H3N2) in MDCK cells. Thirdly, compared with other modeling methods, the network-based method maintained good interpretability of the molecular mechanisms. Combining with systems pharmacology analysis, we highlighted the key influenza host targets and the underlying signaling pathways that may be regulated by BLG, providing a reference for future related studies.

But several limitations of the presented study should be recognized. Firstly, although multiple sources of CTIs, such as experimentally validated and network-based inferred, were imported to construct the CTI network of BLG, incompleteness and imperfections may inevitably exist. Secondly, the network-based models for the identification of active ingredients can prioritize the potential against IAV, which is essentially qualitative rather than quantitative prediction. Thirdly, although previous chemical analysis (Wang et al., 2013) and authoritative herbal medicine databases, such as TCMSP (Ru et al., 2014), ETCM (Xu et al., 2019), TCMIO (Liu et al., 2020), and YaTCM (Li et al., 2018), have confirmed the existence of the predicted compounds in BLG, their exact contents or concentrations in the extract require further detection. Fourthly, the *in vitro* assays revealed that 6 of the 23 predicted candidates showcased efficacy in H1N1- and H3N2-infected MDCK cells; however, their inhibitory activity toward other IAVs, such as H5N1 and H7N9, should be further investigated. Furthermore, the remaining unvalidated compounds also deserved to be studied using experimental assays. Finally, the experiment at the cellular level was merely a preliminary exploration; further in-depth *in vivo* and clinical validations are necessary in a follow-up study.

## Conclusion

This study provides a useful strategy for discovering the active ingredients and exploring the therapeutic mechanisms of BLG against IAV. The network-based framework showcased availability and accuracy for lead identification of anti-influenza compounds, which successfully identified six active candidates after evaluation using *in vitro* assays. On the basis of continuous optimization of the performance of the predictive model by further integrating broader multi-omics data, this study strategy of *in silico* prediction combined with experimental evaluation may serve as a novel and valuable protocol to accelerate the development of novel anti-IAV agents from herbal medicine or a large scale natural product library, but additional in-depth experimental studies are required to further validate the bioactivity and define the molecular of mechanisms.

## Data Availability

The original contributions presented in the study are included in the article/Supplementary Material. Further inquiries can be directed to the corresponding authors.
