# Supplementary figures and images for: In Silico Prediction and Bioactivity Evaluation of Chemical Ingredients Against Influenza A Virus From Isatis tinctoria L
Source: Front Pharmacol. 2021 Dec 7;12:755396. doi: 10.3389/fphar.2021.755396 (PMC8689007; doi:10.3389/fphar.2021.755396)

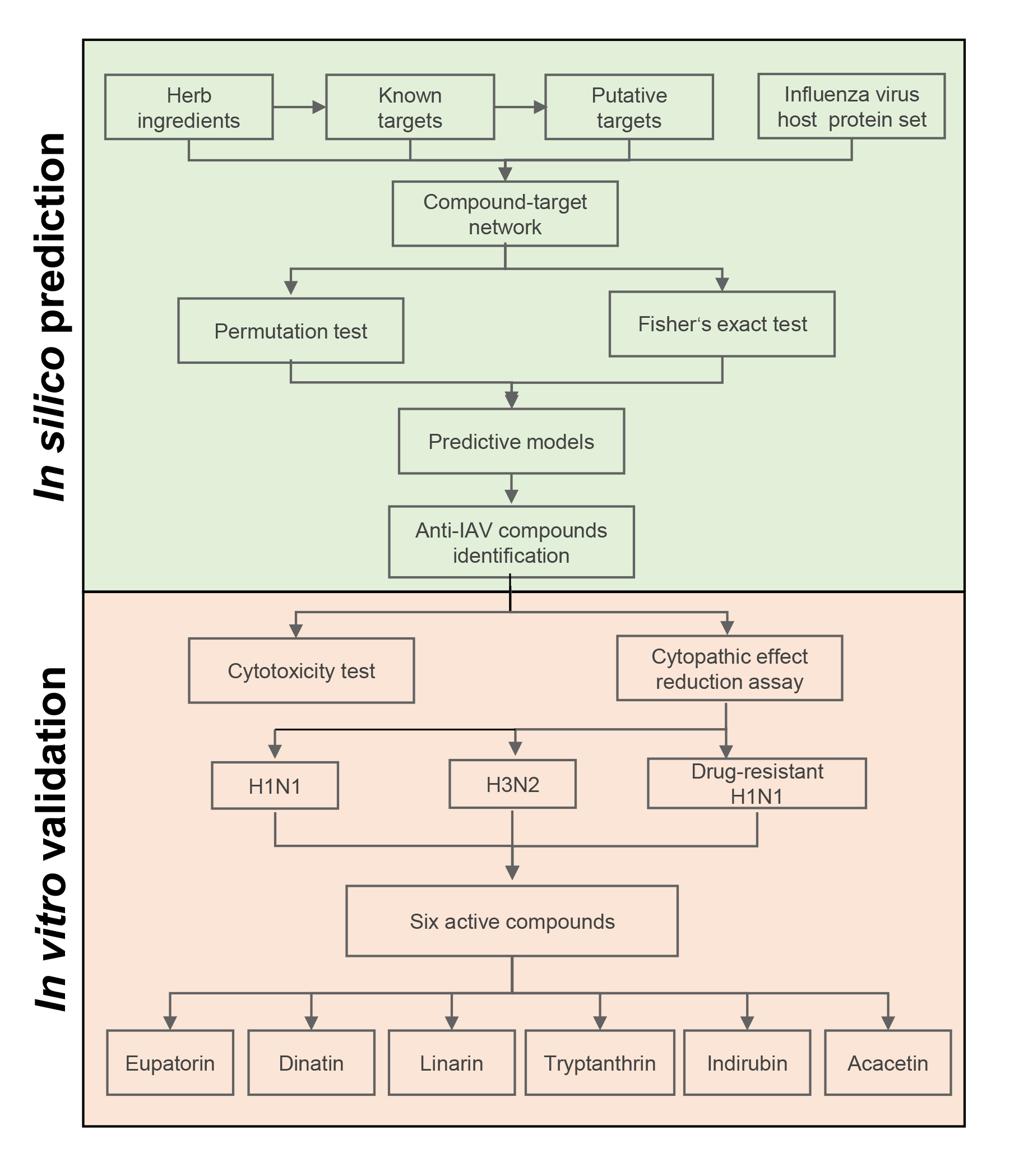

Supplement: Supplementary file 1 [file DataSheet1.ZIP › Supporting information/Figure S1. Workflow of network-based anti-IAV drug discovery combined with experimental-validation from BLG.tif]
